# The Social Effect of “Being Imitated” in Children with Autism Spectrum Disorder

**DOI:** 10.3389/fpsyg.2016.00726

**Published:** 2016-05-13

**Authors:** Annarita Contaldo, Costanza Colombi, Antonio Narzisi, Filippo Muratori

**Affiliations:** ^1^Department of Developmental Neuroscience, IRCCS Stella MarisCalambrone, Italy; ^2^Department of Psychiatry, University of MichiganAnn Arbor, MI, USA

**Keywords:** imitation, being imitated, ASD, early intervention, social brain

## Abstract

There is evidence that “being imitated” has social effects, and that the imitation of the child's actions may be used as a strategy to promote social engagement in children with autism spectrum disorder (ASD). The observation of someone that imitates us recruits, indeed, neural areas involved in social cognition. We reviewed studies exploring the behavioral consequences of “being imitated” in children with ASD. We aimed at assessing what are the social skills targeted by this strategy, and the factors that may improve the response. The “being imitated” strategy improves social gazes, proximal social behaviors, and play skills, particularly in children with low developmental level, and also when the strategy is implemented by children's mothers. The “being imitated” may be used as a tool in early intervention to improve social skills, helping to assess the effects of intervention at both behavioral and neural level.

## Introduction

Imitation is a complex ability that plays a crucial role in social interaction, both in adulthood (Chartrand and van Baaren, 2009), and in earliest infancy (Meltzoff and Moore, 1992). The tendency of adults to unconsciously imitate postures and gestures of a partner, the so called “chameleon effect,” is thought to be a sort of “social glue” that promotes affiliative behaviors (Uzgiris, 1981; Chartrand and van Baaren, 2009) as well as identification within social groups (Lakin et al., 2003). The social effects of imitation affect both the imitator and the imitated subjects (Uzgiris, 1981). Some experimental studies, indeed, showed that after being imitated, people feel closer to others (Ashton-James et al., 2007) and show an increase in the prosocial orientation toward both the imitator and other people (van Baaren et al., 2004).

Also in infancy, “being imitated” promotes a social orientation toward others. From the age of 9 months, infants recognize when others are imitating them (Agnetta and Rochat, 2004). They pay closer attention, and smile more at an adult who imitates their actions compared with one who responds to their actions without imitating (Meltzoff and Moore, 1999; Carpenter et al., 2013). This increase in social attention has been considered an implicit form of imitation recognition (Nadel, 2002). From 14–18 months of age, infants show a more mature form of imitation recognition. After being imitated, they engage in “testing behaviors” (i.e., repeating or varying actions while watching the imitative partner) to test whether the other is imitating them (Meltzoff, 1995; Asendorpf et al., 1996; Nielsen, 2006).

Therefore, very early in development, infants produce imitation and recognize when others are imitating them; these skills represent the two faces of imitation and are both linked to the development of socio-communicative abilities, such as joint attention, intention understanding, and social reciprocity (Carpenter and Tomasello, 1995; Meltzoff, 1995; Nadel, 2002; Tomasello et al., 2005). Through its two faces (imitating and being imitated), the imitation represents a powerful system of communication (Nadel-Brulfert and Baudonniere, 1982; Meltzoff and Moore, 1992; Nadel, 2002). It has been suggested that reciprocal imitation helps infants to understand that they can act like others and that others can act like them (Meltzoff, 2007). According to the Meltzoff's “Like-me” theory, the recognition of being imitated by others is the starting point for social and cognitive development.

This “like me” recognition of others is thought to be rooted in the same neural system underlying imitation: the mirror neuron system (MNS; Bernier et al., 2007; Marshall and Meltzoff, 2014), which includes the posterior part of the inferior frontal gyrus (IFG), the premotor cortex (PM), and the inferior parietal lobe (IPL; Rizzolatti and Craighero, 2004; Dinstein et al., 2007). MNS is activated by both imitation and action observation (Iacoboni et al., 1999; Buccino et al., 2004; Rizzolatti and Craighero, 2004), and plays a key role in understanding the goal or the meaning of an observed action (Buccino et al., 2004; Gallese et al., 2004; Hamilton and Grafton, 2006; Bernier et al., 2007). Thus, the same mechanisms that allow reproduce the actions of another individual might underlie the ability of recognizing when one is imitated (Decety et al., 2002; Nadel, 2002). In addition, a brain network encompassing the medial orbitofrontal cortex/ventromedial prefrontal cortex (mOFC/vmPFC) and the functionally connected striatum and mid-posterior insula, also involved in the processing of emotional or reward-related stimuli, is activated during the observation of another individual that imitates us (Kühn et al., 2010).

Due to its crucial role for social cognitive development, imitation has been extensively studied in children with autism spectrum disorder (ASD), a neurodevelopmental disorder characterized by impairments in social communication and restricted and stereotyped interests and behaviors (American Psychiatric Association, 2013). Imitation skills or propensity to imitate are significantly impaired in children with ASD from the beginning (Rogers and Pennington, 1991; Williams et al., 2004; Vivanti, 2015). However, some evidence suggests that, like typically developing children and adults, they recognize imitation, and respond positively to “being imitated” by an adult partner (Nadel, 2002; Colombi et al., 2009; Field et al., 2011). Therefore, while their ability to produce imitation is impaired, it is possible that their response to “being imitated” is relatively preserved. Some interventions for children with ASD involve the imitation of the child's actions as a strategy to promote social engagement. These behavioral interventions have been demonstrated to be effective in improving imitation skills (McDuffie et al., 2007; Ingersoll, 2008) as well as social abilities (Dawson et al., 2010; Rogers et al., 2012).

However, there are several key points that need better understanding, to evaluate the usefulness of the “being imitated” in early intervention in ASD. First, what are the social skills more strengthened by this strategy? Indeed, the specific effect of “being imitated” on social abilities in children with ASD is unclear. Second, does a relationship exist between chronological and mental age of the child with ASD and the response to “being imitated?” As previously reported, a more mature explicit form of imitation recognition appears later than the implicit form, in typical development. This explicit form, that subtends the ability to recognize, reproduce, and vary the observed actions, has been linked to the development of more advanced social skills. Third, could the specific characteristics of the setting in which the “being imitated” strategy is implemented (i.e., familiarity of the imitator and repeated session of “being imitated”) elicit different responses? Fourth, based on studies exploring the neural basis of “being imitated” in healthy adults and infants, is it possible to hypothesize that this strategy might have a role in promoting the neural reorganization of circuits considered dysfunctional in ASD? The development of social brain is the result of the interaction between the child and the environment, and the engagement with a social partner during reciprocal imitation might promote the cortical specialization of social brain circuitry.

Based on these four questions, this article is organized in three sections (Part 1, Part 2, and Part 3), followed by a brief conclusion section. The first part is an overview on the neural and neurophysiologic correlates of “being imitated” in healthy adults and infants. In the second part, we review the studies exploring the behavioral consequences of “being imitated” in children with ASD and discuss the implications of results for better understanding the link between “being imitated” and social skills in ASD, and the usefulness of the “being imitated” strategy in early intervention. A search was performed in computerized bibliographic databases (PUBMED, WEB of Science) to identify existing literature on the effects of “being imitated” in children with ASD. Search terms included “reciprocal imitation” and/or “being imitated,” and/or “mimicry,” with either “autism” or “ASD” or “Autism Spectrum Disorder.” Additional articles of interest were also located in the reference sections of the articles from the search. Articles included in this review assessed the specific effects of “being imitated” on child's social behaviors. We excluded intervention studies in which the imitation was one of the several naturalistic techniques, such as, for example, studies on the effectiveness of Reciprocal Imitation Training (Ingersoll and Schreibman, 2006; Ingersoll, 2012). In the third part we link behavioral and neuroscientific evidence to discuss the role of “being imitated” in improving both behavior and brain functioning.

## Part 1: the neural basis of being imitated

Regarding the neural basis of “being imitated” two main questions have been addressed in the literature: how our brain detects that we are imitated, and why should we feel better when someone imitates us? Both the awareness of self-other distinction and the social reward deriving from being imitated have been taken into account to answer these questions.

First studies aimed at exploring the neural basis of “being imitated” employed positron emission tomography (PET) to examine the hemodynamic changes produced when subjects imitated the experimenter's actions or when they were imitated by the experimenter (Chaminade et al., 2002; Decety et al., 2002). They found a different lateralization in the inferior parietal lobule (IPL), related to the “imitate” and “being imitated” condition. The left IPL was activated when subjects imitated another person, whereas the right homologous region was associated with “being imitated” condition. Authors suggest that the IPL might play a fundamental role in the sense of agency, i.e., in attributing the generation of an action to self or to other (Decety and Chaminade, 2003) and in distinguishing self-produced actions from those generated by others (Decety et al., 2002; Chaminade et al., 2002). They postulated that, whereas the left IPL computes the sensory-motor associations necessary to imitate, the right IPL is involved in recognizing that the action performed by the other is similar to that initiated by the self (Decety and Chaminade, 2003). Therefore, when subjects observe their actions imitated by another individual, the right IPL might play a key function for the identification with others and for experience intersubjectivity (Decety and Chaminade, 2003).

Many researchers have demonstrated that the observation of actions engages the same brain areas activated when performing similar actions (Iacoboni et al., 1999; Buccino et al., 2004). These areas include the core components of the MNS, the IPL, and the IFG (Rizzolatti and Craighero, 2004). Indeed, the human MNS is thought to play a key role in imitation, action understanding and social cognition (Gallese et al., 2004; Bernier et al., 2007). The function of the MNS is based on a direct-matching model in which observed actions are directly mapped onto the observers own motor system (Rizzolatti and Sinigaglia, 2010). However, mirror neuron properties may be also based on alternative mechanisms. Indeed, the associative sequence learning (ASL) theory suggests that mirror neurons arise from those sensorimotor experiences, including the being imitated, in which there is a contingent or predictive relationship between observed and performed actions (Heyes, 2010).

It has been proposed that MNS may have evolved, sub serving communication, through firstly the imitation of manual actions, and afterwards the imitation of manual gestures used to communicate, and finally the language (Rizzolatti and Arbib, 1998; Arbib, 2005). This hypothesis is supported by evidence showing that the use of imitation to support communication is more frequent when children are not yet able to use language, and seem to decline after language abilities increases (Nadel, 2002). Nagy et al. (2010), in an fMRI-based study, showed the activation of a lateralized network in the MNS during a communicative paradigm of reciprocal imitation in which the subject both imitated the experimenter's movements and elicited an imitation from the experimenter. Differently from a control condition (non-imitative movement), these imitative conditions recruit a lateralized fronto-parietal network, comprising the right IFG and the left IPL. A strong recruitment of parietofrontal regions in the MNS during reciprocal imitation was also found in the Guionnet et al. fMRI study (2012). In this study, a paradigm of online social interaction was employed to explore the patterns of brain activation developed in a real social interaction where two individuals matched their movements as imitator and model. This experiment was composed of three conditions: free imitation, instructed imitation, and observation. Both free and instructed imitation conditions included two subconditions: imitate and being imitated. Authors found a recruitment of parietofrontal regions in the MNS, regardless of the condition (free or instructed imitation) and of the subcondition (imitate or being imitated). However, they found a greater activation in the dorsal part of the anterior cingulate gyrus (dACC), in the left dorsolateral prefrontal cortex (DLPFC), in the dorsal part of the left anterior insular cortex (dAIC) combined with an increased deactivation in the default mode network (DMN), in the being imitated compared to the imitate subcondition. The authors suggested that these patterns of activation when subjects were imitated might reflect the engagement with others required by social interaction (Guionnet et al., 2012).

However, the role of the MNS in action understanding and social cognition was recently reconsidered based on the assumption that a “mentalizing network,” consisting of the temporo-parietal junction (TPJ) and the cortical medial structures (CMS), participates and interacts with the MNS in social understanding (Keysers and Gazzola, 2007; Uddin et al., 2007). Indeed, when “being imitated” has been studied as part of the interaction between two persons, a strong connection between the MNS and the Mentalizing System has been found (Sperduti et al., 2014).

Studies exploring the neural basis of “being imitated” during infancy employed electroencephalographic (EEG) methods during a reciprocal imitation paradigm and focused on the sensorimotor mu rhythm (Reid et al., 2011; Saby et al., 2012). The mu rhythm is considered associated with the activity in the MNS and its desynchronization occurs already in infancy during action execution as well as action observation (Marshall and Meltzoff, 2011). Saby et al. (2012) compared 14-month-old infants' EEG responses during the observation of the same action presented across two different contexts: in one condition, the infants observed the experimenter's action after carrying out the same action, whereas in the other condition they observed the experimenter's action after performing a different action. A greater desynchronization in the mu rhythm was found when infants observed the experimenter imitating their actions than when observing an experimenter's action temporally contingent on the infant's act but non-imitative. The authors stated that the mu rhythm desynchronization during infants' observation of actions is enhanced when there is an imitative connection between the infant's and the observed action (Saby et al., 2012). Moreover, the observation of an experimenter who is attempting to imitate infants' body movements and postures determined greater desynchronization of the 14-month-old infant's mu rhythm compared with a condition in which the experimenter performed a sequence of unfamiliar body movements in a non-interactive fashion (Reid et al., 2011).

All these experimental studies show the activation of multiple brain areas linked to the recognition that the other is imitating us. They do not provide a unified picture and this may be also compatible with the different experimental paradigms employed in these studies. The brain, indeed, processes both the observed action and its social meaning. However, these evidences do not explain fully why “being imitated” promotes prosocial behavior. To answer this question Kuhn and colleagues explored, in an fMRI study, the positive consequences of “being imitated” by means of an observation paradigm in which participants observed an interaction between two actors (Kühn et al., 2010). They found that the observation of a “being imitated” interaction compared to a “not being imitated” interaction activates brain areas that have been associated with emotion, friendship and reward processing, namely medial orbitofrontal cortex/ ventromedial prefrontal cortex (mOFC/vmPFC) (Bartels and Zeki, 2004; Güroğlu et al., 2008). Sharing the same emotional moods and performing the same movements leads to higher levels of activity in brain areas that have been associated with reward processing, but, interestingly, the content of the behavior that is mimicked (i.e., positive or negative emotions) does not seem to play an important role (Kühn et al., 2011).

## Part 2: the behavioral consequences of “being imitated” in children with ASD

The search identified 14 studies that have analyzed the behavioral consequences of “being imitated” in children with ASD. All the studies reviewed are summarized in Table 1. To identify the specific response to “being imitated” we categorized the reviewed articles according to the behavioral measures targeted by the study: social attention (mainly eye gaze behavior), social responsiveness (smiling, verbalizing, vocalizing, approaching, touching toward the experimenter, gestures), motor activities and stereotypies, object manipulation, and play, and imitation skills. As some studies examined multiple measures, the results of a study can be found in different paragraphs. Moreover, to identify the role of both child and experimental setting characteristics in modulating the effect of “being imitated,” we reported the response to “being imitated” in function of the developmental level of the participants and the characteristics of the experimental setting (i.e., the familiarity of the imitative partner, the number of imitative sessions and the type of imitative procedure). Both these factors, indeed, have a crucial role in the planning of intervention strategies.

**Table 1:** **Summary of studies reviewed**.

	**Participants**	**N/Type/Length of session**	**Type of experimental procedure and familiarity of imitative partner**	**Target behaviors**	**Results**
Dawson and Adams, 1984	Autism: *N* = 15 Mean Age = 60.6 months IQ = 17–89 (mean: 57.42) Imitation skills (assessed with Uzgiris-Hunt Developmental Scale) = 8 high, 7 low	1 object play session/20 min	Four conditions: A) Free play B) Simultaneous Imitation C) Familiar Scheme D) Novel Scheme Unfamiliar experimenter	Social attention (looking at the examiner's face) Social responsiveness (smiling at, gesturing, touching, and verbalizing to the experimenter) Object manipulation	Social behaviors were more frequent in Condition B than in D only for low imitators. Children with high imitation skills showed similar response to all conditions. The number of object scheme and toy changes was higher in Simultaneous Imitation than in Novel scheme Condition only for low imitators.
Katagiri et al., 2010	Autism: *N* = 16 Two groups = 2 year-old (23.7 months) IQ = 60–100 (mean 78.4) 3 year-old (37.7 months) IQ = 55–135 (mean 92.3)	1 object play session/7 min	Three phases: BL1: 2-min baseline (the experimenter manipulated toys different from those the child manipulated). MP: 3-min mirroring (the experimenter reproduced everything the child did). BL2: a second 2-min baseline (as in BL1). Unfamiliar experimenter	Social attention (gazing at the experimenter's face) Social responsiveness (smiling, verbalizing, vocalizing, approaching, touching, offering toys) Imitation recognition (requesting the experimenter to imitate his/her own action)	Social attention was more frequent in the MP than in the BL1 phase in the 2-year-old group. Social behaviors were more frequent in the MP than in the BL1 phase in the 3-year-old group.
Berger and Ingersoll, 2013	Autism: *N* = 30 Age = 22–93 months (mean 41.45) MA = mean 23.40 months	1 object play session	During an unstructured imitation assessment (UIA, Ingersoll and Meyer, 2011), the experimenter imitated the child's play with a duplicate toy	Less Mature-Imitation recognition (LM-IR): child's looks at the experimenter toy and face. More Mature-Imitation recognition (MM-IR): Testing behavior and looks plus social signal.	The frequency of MM-IR was lower than that of LM-IR. Significant correlation between MM-IR and object and gesture imitation, and social reciprocity. No correlation between MM-IR, LM-IR and developmental level.
Dawson and Galpert, 1990	Autism: *N* = 15 Age = 2–6 years DL = 7 High, 8 Low	14 object play sessions/20 min	Mothers were instructed to imitate child's actions during each session	Social attention (eye gaze behavior) Object manipulation	The duration of eye gaze behavior increased from pre- to post-intervention. The number of object schemes was higher in post-intervention.
Tiegerman and Primavera, 1981	Autism: *N* = 6 Age = 4–6 years Low DL	17 object play sessions/30 min	Three type of play interaction were presented during each session: A) experimenter imitated the child's action with the same object; B) experimenter performed a different action with the same object; C) Experimenter performed a different action with a different object	Object manipulation	The frequency and duration of object manipulation was higher in Condition A than in B and C. The frequency and duration of object manipulation increased significantly over sessions under condition A and this increase was greater than that under condition B and C.
Tiegerman and Primavera, 1984	Autism: *N* = 6 Age = 4–6 years Low DL	17 object play sessions/30 min	As in Tiegerman and Primavera, 1981	Eye gaze behavior	The frequency and mean duration of eye gaze behavior was higher in Condition A than in B and C. The frequency and duration of gaze behavior increased significantly over sessions under condition A and B, and this increase was greater than that under C.
Nadel et al., 2000	Autism: *N* = 8 Age = preschooler Low DL	1SF session /12 min	*SF modified paradigm:* four phases SF1: First still-face IP: Imitation phase SF2: Second still-face SP: Spontaneous play phase Unfamiliar experimenter	Social attention (looking at a person) Social responsiveness (positive facial expressions, negative facial expressions, social gestures, close proximity, touch).	The frequency of looking at a person, negative facial expressions, social gestures, close proximity, and touch behaviors was significantly higher in SF2 than in SF1 after the imitation phase
Escalona et al., 2002	Autism: *N* = 20 Age = 3–7 years Low DL Two groups: Imitation (*N* = 10) Contingent (*N* = 10)	1 SF session/12 min	*SF modified paradigm*: see above The second phase was different in two groups (imitative or contingently responsive) Unfamiliar experimenter	Social Attention (looking at adult's face) Motor activity and stereotypies Social responsiveness (silence, touching, distance from adult)	The frequency of motor activity was lower and the frequency of touching behavior was higher in SF2 vs. SF1 in the Imitation group. A reduction in distance from the adult was observed in both groups. A decrease in silence (or increase in sounds) was observed in the Contingent group. The frequency of looking at adult was higher in SF2 vs. SF1 only in the Contingent group. No effect on stereotypies.
Field et al., 2001	Autism *N* = 20 Age = 4–6 years Low DL Two groups: Imitation (*N* = 10) Contingent (*N* = 10)	3 SF sessions/12 min	*SF modified paradigm*: see above The second phase was different in two groups (imitative or contingently responsive) Unfamiliar experimenter	Social attention (looking at adult) Social responsiveness (vocalizing/smiling, being close/touching adult, engaging in reciprocal play) Autistic behaviors (stereotypies, playing alone, inactivity) Object play behavior Imitation recognition	The frequency of looking, vocalizing/smiling, touching, and engaging in reciprocal play increased from pre- to post intervention more in the Imitation than in the Contingent group. A reduction in Autistic behaviors (playing alone and inactivity), an increase in Object play behavior and in imitation recognition were observed from pre- to post-intervention more in the Imitation than in Contingent Condition group.
Field et al., 2013	Autism *N* = 20 Age = 4–6 years Low DL Two groups: Imitation (*N* = 10) Contingent (*N* = 10)	3 SF sessions/12 min	*SF modified paradigm*: see above The second phase was different in two groups (imitative or contingently responsive) Unfamiliar experimenter	Social attention (looking at adult) Novel object play behaviors Imitation skill	Children in the Imitation group vs. those in the Contingent group showed higher frequency of looking and novel object play behaviors during the imitation phase. Children in the Imitation group showed higher frequency of looking and imitation behaviors than those in the contingent group in the SP phase.
Heimann et al., 2006	Autism: *N* = 20 Age = 4:4–12:9 years MA: 1:0–4:5 years (mean 2.1) Two groups: Imitation (*N* = 10) Contingent (*N* = 10)	2 SF sessions /12 min	*SF modified paradigm*: see above The second phase was different in two groups (imitative or contingently responsive) Unfamiliar experimenter	Social attention and responsiveness (Touch, Look and Request) Imitation skill	Significant increase in Social behaviors from SF1 to SF2 for children in the Imitation Condition group, but not for children in the Contingent Condition group. A significant increase in Social behaviors was also found in the SP phase after the second session.
Sanefuji and Ohgami, 2011	Autism: *N* = 32 Age = 54.38 months MA = 27.91 months Two groups: Imitation (*N* = 16) Contingent (*N* = 16) TD: *N* = 32 Age = 27.41 months Two groups: Imitation (*N* = 16) Contingent (*N* = 16)	1 SF session/8 min	*SF modified paradigm*: addition of a spontaneous play phase before SF1 The intervention phase was different in two groups (imitative or contingently responsive) Mother	Social attention (child's looking time directed to the mother)	Children with TD looked at their mothers for a longer duration than did those with ASD. No differences in the frequency of gaze behavior between two groups (imitation and contingent) Children with autism in the Imitation group showed higher frequency of gaze behavior in the SF2 vs. SF1 and in the Imitation phase.
Sanefuji and Ohgami, 2013	Autism: *N* = 16 Age = 53.63 months MA = 52.63 months Two groups: Imitation (*N* = 8) Contingent (*N* = 8)	Five minutes to day for a 2-months period	A SF session (as in Sanefuji and Ohgami, 2011) was conducted before and after the home-based intervention. The intervention was different in two groups (imitative or contingently responsive) Mother	Social attention Object actions imitation	After intervention, children in the Imitation group looked at their mothers for a longer duration and imitated object actions more than did children in the Contingent group.
Slaughter and Ong, 2014	Autism: *N* = 10 Age = 2 years, 8 months to 8 years, 4 months; MA = 5 years, 5 months	2 SF session/9 min	*SF modified paradigm*: addition of a spontaneous play phase before SF1 Children took part in two sessions: one with their mother and the other with the unfamiliar experimenter	Social attention (Looking toward the adult) Social responsiveness (vocalization directed to the adult, approaching, touching, social gestures) Play skills	A significant increase in looking and vocalizing toward the adult was observed in the SP2 phase vs. SP1 with both partners. A significantly greater increase in proximal social behaviors and a greater decrease in playing alone were observed in SP2 when the imitator was the child's mother as opposed to the experimenter. No significant increase in social gestures was found. A significant decrease in playing alone was observed in SP2 after imitation by the mother.

*N, number; IQ, intelligence quotient; MA, mental age; TD, typical development. DL, developmental level; SF, still face*.

Studies investigating the behavioral consequences of “being imitated” used two different experimental procedures to evaluate the effects on social cognitive abilities. Six studies employed an experimental paradigm in which an unfamiliar experimenter or the child's mother copies the child's object-directed actions, gestures, and vocalizations during a single (Dawson and Adams, 1984; Katagiri et al., 2010; Berger and Ingersoll, 2013) or repeated object play session (Tiegerman and Primavera, 1981, 1984; Dawson and Galpert, 1990).

Another series of studies (Nadel et al., 2000; Field et al., 2001, 2013; Escalona et al., 2002; Heimann et al., 2006; Sanefuji and Ohgami, 2011, 2013; Slaughter and Ong, 2014) investigated the effects of “being imitated” using the Nadel et al. (2000) adapted version of the Still Face (SF) paradigm (Tronick et al., 1978). In the adapted SF paradigm, children with autism interacted with an adult for four phases, each lasting 3 min: First still-face (SF1), Imitation Phase (IP), Second still-face (SF2), and Spontaneous Play (SP). During the SF1, the child walked into a room that was furnished with a sofa, a table, chairs and two sets of identical toys. An unfamiliar adult sat on the sofa with a still face and did not move. During the following IP, the adult imitated everything the child did including the autistic behaviors, such as motor stereotypies and repetitive actions with objects. The SF2 was similar to the first one, and the fourth phase was a spontaneous interaction in which the adult played freely with the child (Nadel et al., 2000). Nadel et al. (2000) hypothesized that, if children looked more at the adult in the SF2 with respect to SF1, after IP, they had developed social expectancies toward the adult. Therefore, to explore the effects of “being imitated” on social responsiveness in children with ASD, three studies compared the child's behavior between the SF1 and SF2 phases (Nadel et al., 2000; Field et al., 2001; Escalona et al., 2002), whereas five studies analyzed the child's behaviors also during the IP and the SP phases (Heimann et al., 2006; Sanefuji and Ohgami, 2011, 2013; Field et al., 2013; Slaughter and Ong, 2014). These latter authors hypothesized that an increase of social behaviors in SP phase after imitation could indicate a generalization of the effects. The results of these studies are, therefore, crucial in order to identify strategies that can be truly effective on social behavior. Children with ASD, indeed, are known to have difficulty in generalizing recently acquired skills to new environments (Ozonoff and Miller, 1995). As for the object-play situation, also in the SF situation the session was single (Nadel et al., 2000; Escalona et al., 2002; Sanefuji and Ohgami, 2011; Slaughter and Ong, 2014) or repeated (Field et al., 2001, 2013; Heimann et al., 2006; Sanefuji and Ohgami, 2013), and was administered by an unfamiliar experimenter (Nadel et al., 2000; Field et al., 2001, 2013; Escalona et al., 2002; Heimann et al., 2006) or by the child's mother (Sanefuji and Ohgami, 2011, 2013; Slaughter and Ong, 2014).

In six studies, the effect of “being imitated” on social behaviors was compared to the effect of a contingent interaction, in which the social partner responds immediately to the child with a similar but not imitative behavior (Field et al., 2001, 2013; Escalona et al., 2002; Heimann et al., 2006; Sanefuji and Ohgami, 2011, 2013). This latter form of interaction, indeed, had been recognized as a useful strategy to promote engagement in ASD.

In the reviewed studies the behavioral measures targeted were: (a) social attention (eye gaze behavior), (b) social responsiveness (distal social behaviors, as smiling, verbalizing; proximal social behaviors as approaching, touching; social gestures as pointing, requesting, offering, and showing), (c) motor activity and stereotypies, (d) object manipulation and play and (e) imitation skills.

### Social attention

One of the core symptoms of ASD is the presence of early deficits in social attention, and in establishing and maintaining eye contact. Some authors hypothesized that the early atypical pattern of attention preclude social input that normally promotes the development of social and linguistic brain circuitry during early sensitive periods (Dawson, 2008). For this reason, understanding what strategies are useful to strengthen the visual social attention is crucial for early intervention.

The eye gaze behavior was targeted by most of the reviewed studies. Using the SF paradigm, Nadel et al. (2000) found that children with ASD recognize the adult's imitation, and look more at him/her in the SF2 phase, after being imitated. This effect appears dependent on the imitative interaction with the other and it is not due to a contingently responsive interaction with a partner. Six studies, indeed, compared the effects of an imitative vs. a simply contingently responsive interaction, by employing a SF procedure in which the second phase could be either imitative (Imitation Condition, IC) or contingently responsive (Contingent Condition, CC) (Escalona et al., 2002; Heimann et al., 2006; Field et al., 2011, 2013; Sanefuji and Ohgami, 2011, 2013). All but one studies (Escalona et al., 2002) found that the frequency and duration of eye gaze behavior toward the adult was higher after the imitation than after the contingent condition. Escalona et al. (2002), using the same paradigm, found that the looking time at the adult did not change in the Imitation group but showed an increase only in the Contingent group. In the Heimann et al. study (2006) the percentage of time that the children displayed “looking at a person” behavior was combined with the “touching” and “requesting” behaviors in a Social Interest composite score, so we cannot know the specific increase in eye gaze behavior. However, Social Interest Score increased in SF2 vs. SF1 in the Imitation but not in the Contingent group (Heimann et al., 2006). This effect was evident for both the SF phase following directly the imitation phase and the SP phase at the end of each session. Also other studies (Field et al., 2013; Sanefuji and Ohgami, 2013; Slaughter and Ong, 2014) found an effect on social attention during a spontaneous play phase after being imitated. This could indicate a possible generalizing effect. No study, however, tested whether the increase in social attention after being imitated extends to persons other than the imitator.

A different pattern of response to imitative vs. contingent interaction with the mother was found in typical development (TD) children with respect to children with ASD (Sanefuji and Ohgami, 2011). Children with ASD, indeed, looked at their mothers longer in the Imitative than in the Contingent condition whereas children with TD looked at their mothers longer than those with ASD, but without differences between the two conditions (Sanefuji and Ohgami, 2011). Therefore, the interaction pattern that is able to determine a social effect in children with ASD could be different with respect to children with TD. In children with ASD, indeed, a greater social effect might be determined by those interactions that are characterized by perfect, more than imperfect, contingency; in the “being imitated” strategy, the contingency is perfect because both the temporal and structural aspects of the action are matched. Recent evidence on healthy adult subjects underlines the greater importance of contingency, regardless of similarity, in producing social effects after being imitated (Catmur and Heyes, 2013). These Authors suggested that the ability in detecting and predicting that our own actions cause the action of another person could engender the social behaviors. It could be therefore hypothesized that the impairment in predictive abilities in children with ASD might determine a lower responsivity to simple contingency. This specific research hypothesis would deserve further attention and experimental data. Autism has been in fact also proposed as a disturbance of prediction (Brown and Brüne, 2012; Pellicano and Burr, 2012). So, one of the reason for which the imitation might be more salient than the contingency is that the former is more predictable and familiar for children with ASD, and requires less anticipatory skills.

Employing a different experimental procedure, Tiegerman and Primavera (1984) found an effect of “being imitated” on social attention during the imitative session. These authors, indeed, compared the effects of imitating the child's actions with the same object during repeated object play sessions with two others different non-imitative interaction procedures: performing a different action with the same object or performing a different action with a different object. They found that frequency and duration of eye gaze behavior were higher during the first interaction procedure than others non-imitative interactions. They also found that the frequency and mean duration of gaze behavior increased significantly over repeated sessions for both the first and second interaction procedures, and this increase was greater than that for the third procedure (Tiegerman and Primavera, 1984). A further consideration may arise from these findings. While an imitative interaction, characterized by a strictly contingency (the same action with the same object at the same time) is able to determine an effect immediately, a non-imitative interaction in which the examiner uses the same object (at the same time) is able to determine the same effect but after repeated sessions. Although the Authors do not deal with this hypothesis, it could be possible that also the contingent use of the same object might be able to increase the visual attention in children with ASD. Indeed, children might have been attracted by the same object in the first sessions and then they might have been realized that their own action had caused the other's action. This predictive relationship between the child's actions and those of the examiner could have contributed to social behavior. Unfortunately, following Tiegerman and Primavera's work (1984), no additional studies compared these two different procedures. Further, research would be needed to establish whether the use of a same object during repeated play interactions could be a useful tool in early intervention.

Moreover, social attention increased after repeated sessions of “being imitated,” both using the SF paradigm (Field et al., 2001, 2013; Sanefuji and Ohgami, 2011) and an object play experimental procedure (Tiegerman and Primavera, 1984; Dawson and Galpert, 1990). Field et al. (2001) performed three sessions using the SF paradigm and found that the time spent for looking the adult increased from pre- to post-intervention more in the SF2 subsequent to the Imitation than to the Contingent condition. Social attention was also greater during the Imitation phase and in SP phases after Imitative with respect to Contingent condition (Field et al., 2013). A significant correlation was found between the percentage of time during which the adult imitated the child during the imitative phase, and the time during which the child showed social attention in the same phase (Field et al., 2013).

After a parent-based intervention, that was either imitative or contingent, Sanefuji and Ohgami (2013) found a greater increase in social gaze in the imitation group with respect to the contingent group. Therefore, the greater effect of imitation vs. contingency on social attention was evident also when the child's mother was the imitative partner. In their study, Dawson and Galpert (1990) found such effect after a child-mother imitative interaction. They observed a higher duration of children gaze during an imitative vs. free play session, and an increase of this effect after a 2-week period during which children engaged in imitative object play with their mother for 20 min per day. In this study, the increase in social attention after being imitated was not correlated to the developmental level of imitation abilities, play skills, Vineland social age, IQ, or severity of autistic symptoms. Slaughter and Ong (2014) examined whether the familiarity with the social partner might modulate the effect of “being imitated” using a SF procedure (Slaughter and Ong, 2014). The children's social behaviors were coded prior to, and following a 3-min period in which an adult imitated everything they did. In one condition, the partner was the child's mother, and in the other condition, the partner was an unfamiliar experimenter. The results revealed significant increases in social attention (gazes toward the adult) and responsiveness (distal social behaviors) in the SP phase following imitation by both partners. This finding supports the engagement of the family as an important aspect of early intervention in ASD (Rogers et al., 2012; Estes et al., 2013).

Regarding the relationship between the developmental level of children with ASD and the effect of being imitated on social attention, one study found no correlation between the effect of “being imitated” on social attention and child development level (Dawson and Galpert, 1990). Conversely, other two studies seem to suggest that the lower is the developmental or chronological age of the toddler with ASD, the greater is the mirroring effect on social attention (Dawson and Adams, 1984; Katagiri et al., 2010). In the Dawson and Adams study, children with ASD were exposed to four interactive procedures: (1) Free-Play Condition, (2) Simultaneous Imitation Condition (the experimenter simultaneously imitated all children's actions), (3) Familiar Scheme Condition (the experimenter modeled an action that is known to be in the child's behavioral repertoire), and (4) Novel Scheme Condition (the experimenter modeled a novel action). Only in children with low imitative abilities (tested with the Uzgiris-Hunt Scale), the frequency and duration of the eye contact behavior toward the experimenter was higher when he/her modeled an imitative action rather than a familiar or novel action. Conversely, children with more developed imitation skills showed similar responses to all conditions (Dawson and Adams, 1984). An increase in the frequency of social attention (eye gaze behavior) during the imitative vs. the free play phase of an object play session was observed in the Katagiri et al.'s study in a sample of children with ASD. This effect was higher in children aged two with respect to a 3-year-old group, and it correlated negatively with the IQ but not with the severity of autistic symptoms (Katagiri et al., 2010). Some considerations, concerning the link between the effect of “being imitated” and the developmental level of children with ASD, deserve to be further discussed. The finding of a greater effect on social attention in low functioning children could be explained as the manifestation of an implicit form of imitation recognition that is present in children with ASD and determines an increase in the frequency of gaze toward the other but not an increase in more advanced social skills. In support to this hypothesis, findings from Berger and Ingersoll work (2013) demonstrate that a less mature form of imitation recognition is more frequent than a more mature form. Moreover, more advanced social behaviors after “being imitated,” as offering toys to the experimenter and requesting the experimenter to imitate his/her own action, correlate positively with the age and the developmental level (Katagiri et al., 2010). Accordingly, children with higher imitation ability and higher developmental and chronological age might show a smaller effect on visual attention because they respond to being imitated with more mature social behaviors. However, the increase in visual attention, showed by low functioning children, could be the demonstration of the capacity to perceive, during highly predictive interactions, the contingency between the own behavior and that of another person.

Taken together, all studies found an increase in social attention, both during and after the imitative session. However, more studies are needed to evaluate the child characteristics and the type of procedure that are able to modulate this effect.

### Social responsiveness

The effects of “being imitated” on other social behaviors than eye gaze were analyzed in eight studies (Dawson and Adams, 1984; Nadel et al., 2000; Field et al., 2001, 2013; Escalona et al., 2002; Heimann et al., 2006; Katagiri et al., 2010; Slaughter and Ong, 2014). These behaviors included smiling and verbalizing to the adult (distal social behaviors), touching and approaching the adult (proximal social behaviors), engaging in reciprocal play, and producing social gestures.

Dawson and Adams (1984) reported that only the children with low imitation abilities showed a frequency of proximal and distal social behaviors that was higher during a Simultaneous imitation, than during a Novel condition. Children with high imitation skills showed similar response to all conditions. Therefore, the “being imitated” strategy might be a useful tool for children with ASD, in particular those with low developmental level. On the other hand, in children with higher imitation and developmental abilities, a less predictive interaction, as a non-imitative but contingent procedure, could result in the same social effect than an imitative procedure.

A significant increase in social responsiveness in children with low functioning was also found in other studies using a SF paradigm (Nadel et al., 2000; Field et al., 2001; Escalona et al., 2002; Heimann et al., 2006). They reported an increase in proximal social behaviors during the SF2 after imitative session. Escalona et al. (2002) reported that social proximal behaviors increased in the Imitation group with respect to the Contingent group. Moreover, a decrease in the distance from the adult was found in both the imitation and contingent groups (Escalona et al., 2002). In the studies of Field et al. (2001, 2013) children showed an increase in both distal and proximal social behaviors, and in engaging in reciprocal play with the experimenter, after repeated imitative sessions.

Katagiri et al. (2010) analyzed the effect of being imitated both on Social attention and Social responsiveness (smiling, verbalizing, vocalizing, approaching, touching, offering toys, and requesting the experimenter to imitate his/her own action). Social behaviors were observed in older children (3-year group) more frequently during the imitative than the free play phase of object play session. This effect was not significantly related to the severity of autistic symptoms, but correlated positively with the IQ. So, differently from the effect on social attention, this study showed that more mature social behaviors were more frequent in older and higher functioning children. The inconsistency of this last result with the previous can be explained by the different type of analyzed behaviors. In this study, in fact, social responsiveness includes more mature social behaviors, namely offering toys and requesting the experimenter to imitate his/her own action. Berger and Ingersoll (2013) found that all children with ASD engaged in less mature recognition behaviors (child's looks at experimenter's face and/or toy) in response to imitation. However, a more mature imitation recognition (child's looks, and testing behaviors) was less frequent and fewer children displayed this behavior. Moreover, the authors found a significant relationships between more mature imitation recognition and other social-cognitive skills (social reciprocity, object imitation, and gesture imitation) (Berger and Ingersoll, 2013).

Slaughter and Ong (2014) explored the role of the familiarity of the imitative partner in modulating the effect of “being imitated” on social behaviors. They found a significant increase in proximal social behaviors and a greater decrease in playing alone during the SP phase when the imitator was the child's mother as opposed to the experimenter. Therefore, differently from the social attention that increased with both social partners, the effect of being imitated on proximal social behaviors was modulated by the familiarity (Slaughter and Ong, 2014). This finding highlights the benefits of asking caretakers to imitate their children during early development, in particular in children with ASD.

Overall, these studies report an increase in social responsiveness both during and after the imitative session. Three studies, which have analyzed separately the social behaviors, found a greater increase in proximal behaviors, compared to distal social behaviors. Conversely, the remaining studies had included the proximal and distal social behaviors in a single category. Such methodological aspects, therefore, limit the possibility to address the issue.

But why should the “being imitated” have a positive effect on social behavior? As suggested by Nadel, the experience of being imitated makes the social situation more salient for children with ASD; this might increase the likelihood that these children will show social responses (Nadel, 2002) and the experience of being imitated could strengthen the circles of reciprocal communication. Such reciprocal communication supporting the first non-verbal interactions between newborns and their mothers plays a constitutive role in the development of an implicit sense of self as social agent (Rochat and Striano, 1999; Nagy and Molnar, 2004). This form of reciprocal mother-infant “protoconversation” could be early impaired in children with ASD and, therefore, interfere with the subsequent maturation of social skills. Through reciprocal imitation (imitating and being imitated), children understand the self-other similarity (Meltzoff, 2007). As hypothesized by Meltzoff (2007), indeed, deficiencies in this “like-me” mechanism, that is the foundation of social cognition, could explain the social impairment observed in ASD.

### Motor activity and stereotypies

Only two studies analyzed the effect of “being imitated” on motor activity and stereotypies (Field et al., 2001; Escalona et al., 2002). Escalona and colleagues reported that the imitation condition was more effective than the contingent condition in reducing the time spent in motor activity (running, walking, and jumping). The change in motor activity after the imitation condition was attributed to a greater awareness of the adult that diverted the child attention from motor activity (Escalona et al., 2002). Field et al. (2001) found a reduction in autistic behaviors (inactivity and playing alone) from pre- to post-intervention, more in the imitation than in the contingent group. These results fit with the findings that “being imitated” increases proximal social behavior and visual attention toward the adult. No effect on motor stereotypies was found in both studies and further research is needed to explore this area. Also, since some evidence supports the hypothesis that motor stereotypies may be related to poor motor control in people with ASD (Radonovich et al., 2013), future studies should hopefully test also the effect of “being imitated” on motor stereotypies in relation to motor development.

### Object manipulation and play skills

Along with a persistent deficit in social communication and social interactions, a restricted and repetitive pattern of behavior, interest or activity is the other core diagnostic domain of ASD (American Psychiatric Association, 2013). Five studies analyzed the effect of “being imitated” in reducing this behavioral pattern. The number of object and toy schemes, the frequency of object manipulation, the play skills, and the ability to initiate new behaviors were taken in consideration (Tiegerman and Primavera, 1981; Dawson and Adams, 1984; Dawson and Galpert, 1990; Field et al., 2001, 2013).

More frequent scheme and toy changes were observed during the imitative than the non-imitative interaction in children with low imitative ability, but not in children with high imitative ability that responded in the same way to both conditions (Dawson and Adams, 1984). The increase in frequency and duration of object manipulation was not correlated to the similarity of the objects, but to the behavioral similarity between the child and adult action. Indeed, it was found a higher increase during a play session in which the experimenter imitated the child's actions with the same object, than when he/she performed a different action to the same object or a different action to a different object (Tiegerman and Primavera, 1981). An increase in the number of action schemes performed with the objects was also found after repeated sessions of imitative intervention by children's mothers (Dawson and Galpert, 1990) or unfamiliar adult (Field et al., 2001, 2013). After repeated sessions, an increase in the time spent in playing with objects and in initiating novel play behaviors was found when children were imitated, with respect to the contingent interaction with an adult (Field et al., 2013).

Two considerations emerge from these studies. First, the increase in object schemes and play skills after the “being imitated” intervention could be indicative of the ability of children with ASD of learning through observation. The greater attention and proximity to the other, due to the “being imitated” intervention, could indeed improve the children's ability in playing and reproducing the observed actions. This fits with the idea that boosting social attention ought to enhance performance in social cognition (Chevallier et al., 2012). A second consideration concerns the role of the familiarity of social partner in modulating the effect of “being imitated” on play skills (Dawson and Galpert, 1990; Slaughter and Ong, 2014). Some naturalistic studies, indeed, had underlined the role of infant-parent interactions in supporting the development of social cognitive skills (Vigotsky, 1978; Arbib et al., 2005; Koterba and Iverson, 2009) and in promoting the functional actions with objects (Contaldo et al., 2013), especially through the enhancement of infants' attention. The attention-focusing quality of infant-directed input offers, indeed, to the developing infant increased opportunity to absorb information from the environment. Moreover, these interactions give children the opportunity to discover the object “affordances” relevant for the action with objects (Von Hofsten, 2004; Arbib et al., 2005). The familiarity of an adult during the “being imitated” interactions could enhance, in children with ASD, the effect on object play abilities.

### Imitation skills

There is evidence that people with ASD have a consistent impairment in imitation (Rogers and Pennington, 1991; Williams et al., 2001; Mostofsky et al., 2006). However, the imitation deficit is not global but some skills, such as the imitation of a goal-directed action on an object, are preserved (Rogers, 1999; Williams et al., 2004). In this paragraph, we report evidences of the ability of children with ASD in recognizing the other's imitation and the effect of being imitated on imitation production.

As proposed by Nadel (2002) there are two levels of imitation recognition: at a low level, it consists of the capacity to recognize structural and temporal contingencies without any attribution of imitative intentionality to the imitator; higher levels imply the recognition of the other's intention to imitate. While the former might lead to increased visual attention, the higher level of imitation recognition might lead to behaviors testing whether the other is imitating. The increase in social attention and in proximal behaviors after being imitated, found in most studies, could indicate a low level form of imitation recognition, as children looked more at an adult imitating them than at one performing a contingent but not imitative behavior. Anyway, the increase in gaze toward the imitator did not imply the children awareness of the real intention of the adult to match their behavior (Nadel et al., 2000). A more mature form of imitation recognition emerges when children perform testing behaviors (i.e. repeating and varying actions while watching the imitative partner) to test whether the other is imitating them, or in the presence of more social signal (Asendorpf et al., 1996; Nielsen, 2006). Three studies reported these behaviors denoting a more mature form of imitation recognition (Field et al., 2001; Katagiri et al., 2010; Berger and Ingersoll, 2013). Katagiri et al. (2010) included “requesting the experimenter to imitate his/her own action” in social behaviors and found that the number of the behaviors included in this category increased after being imitated especially in high functioning children. In this study, however, we cannot assess the role of the development level in modulating the effect of being imitated on imitation recognition because such behavior is not assessed separately but within a broader category of social behaviors. The study by Berger and Ingersoll was aimed at measuring the frequency of two different types of imitation recognition during a naturalistic imitation task: (1) less mature imitation recognition (child's looks at experimenter's face and/or toy), and (2) more mature imitation recognition (child's looks plus testing behaviors). They found that all children showed an increase in less mature imitation recognition, while testing behaviors were less frequent, and fewer children displayed this behavior (Berger and Ingersoll, 2013). The authors found no correlation between imitation recognition and developmental level, but the more mature imitation recognition significantly correlated with imitation production abilities. It is a major achievement that makes us think about the relationship between imitation and motor skills. In fact, the more mature imitation recognition is characterized by the ability to reproduce and vary the observed actions (testing behaviors). Both “testing behaviors” and the spontaneous selection of a movement in order to maintain a reciprocal imitation with a partner require stronger predictive ability and motor skills that appeared affected in autism (Esposito et al., 2011; Gowen and Hamilton, 2013; Trevarthen and Delafield-Butt, 2013; Sacrey et al., 2014). In addition, the selection of a movement to elicit a reciprocal imitation appears to be underpinned by the long-distance connections between frontal cortex and parietal regions (Guionnet et al., 2012) that are thought to be particularly affected by the dysfunctional connectivity characterizing ASD (Belmonte et al., 2004; Hoppenbrouwers et al., 2014). Thus, the early difficulties in initiating and maintaining a communication with the caregiver through reciprocal imitation may be linked to a primary motor impairment that is supposed to characterize early autism. If predictive ability and motor skills are lacking in autism, affected children can recognize the imitation of the others, and can be attracted by it, but they are not able to expand it toward higher levels of imitation recognition.

The findings of these studies suggest that children with ASD are able to recognize imitation by others. However, the imitation recognition ability is not completely preserved and this result may be related to motor impairment. Anyway, even if it is indicative of just a basic imitation recognition, the increase of visual attention is very important, as it is target behavior in many models of treatment for ASD.

Four studies investigated the effect of being imitated on imitation production (Field et al., 2001, 2013; Heimann et al., 2006; Sanefuji and Ohgami, 2013). Field et al. (2001) found an increase in mirror play after three sessions of imitative intervention. Moreover, they found that the percent of time spent by children in imitating the adult in the spontaneous play phase was greater after the imitative than the contingent intervention (Field et al., 2013). An increase in imitation production after an imitative vs. contingent intervention was also found by Sanefuji and Ohgami (2013) during a home-based intervention in which mothers were instructed on how to imitate their child's behaviors, including facial expressions and meaningless utterances. The number of actions reproduced after the experimenter's demonstration was coded pre and after intervention: after intervention, children in the Imitation condition imitated object actions more than did children in the Contingent condition. Another study investigated whether “being imitated” had effects on imitation skills elicited outside the experimental paradigm (Heimann et al., 2006). Therefore, elicited imitation was measured using the PEP-R (Psychoeducative Profile-Revised; Schopler et al., 1990), and scores were compared before and after an intervention session that could be randomly imitative or contingent. There were no statistically significant differences in the PEP-R imitation sub-scale scores between the two groups, either before or after the experiment. However, an analysis of the changes in imitation scores revealed that eight out of ten children in the imitative condition increased their scores at post-assessment, while the same result was found in only two children in the contingent condition (Heimann et al., 2006). Such studies might indicate that being imitated has a greater effect on spontaneous imitation compared to elicited imitation. As previously reported, impaired predictive and motor skills could also reduce the effect of being imitated on elicited imitation. Instead, the spontaneous reproduction of familiar actions could be simpler because it requires lower predictive abilities. Moreover, some neuroscientific studies reported different brain activation pattern during elicited vs. free imitation tasks (Guionnet et al., 2012).

The finding that the basic response to “being imitated” is relatively preserved in children with ASD, while their imitation skills are more impaired, could have different possible explanations. First, even at a neural level, imitating and being imitated seem to have a different lateralization in the brain. Indeed, the left IPL computes the sensory-motor associations necessary to imitate an action demonstrated by the other, and the right IPL is involved in recognizing that the action performed by the other is similar to that initiated by itself (Decety et al., 2002; Decety and Chaminade, 2003). Second, the basic level of imitation recognition is cognitively simpler than the imitation production; it requires the matching between others' and self-produced actions (Nadel, 2002). Differently, the imitation production relies on memory capacities as well as on planning and action selection skills.

## Part 3: the social effect of “being imitated” in children with ASD and its role in early intervention

The findings of the behavioral studies previously reported suggest that the imitation of children's actions by therapists or parents, especially at an early age and in children with low developmental and imitation abilities, could be an effective tool to improve social gazes orienting, proximal social behaviors (touching and approaching the adult), and play skills. These effects are evident both during and after the imitative sessions and they increase after repeated imitation sessions. Moreover, these effects are more evident in the “being imitated” condition than in the contingently responsive interaction. Thus, the child's imitation *per se* seems to be responsible for the effect on social behavior. This last finding suggests that the behavioral similarity between the actions of children and the following action of an adult, more than its contingency, should be used as a strategy that would deserve to be systematically included in interventions for children with ASD.

However, the significant interest leading to the design of more effective interventions supposes an effect on neural basis in ASD. Nevertheless, two main theories have been implicated. First, the “broken mirror” theory of autism proposed that a dysfunction of the MNS is responsible for the core social deficits in individuals with ASD (Williams et al., 2001; Dapretto et al., 2006; Oberman and Ramachandran, 2007). However, recent studies suggest that at a neural level, individuals with ASD may not have a global MNS impairment, but they may have impairments within certain nodes of the MNS or in their anatomical connectivity and functional synchronization (Hamilton, 2008; Kana et al., 2011). The evidence that the imitation deficit in autism is not global, but some imitation skills, such as the imitation of a goal directed action on an object, are preserved supports this view (Rogers, 1999; Williams et al., 2004). Such a picture, in which the imitative skills occur with a pattern characterized by peaks and troughs, would seem more compatible with the hypothesis of an alteration in functional connectivity between areas of the nervous system rather than with the hypothesis of a single dysfunction in a “broken” system. Thus, it is supposed that specific training on “being imitated,” could improve MNS dysfunctional area and brain connections with other neural network involved in social cognition.

It has been suggested, indeed, that mirror neurons may develop their sensorimotor properties as a result of experience, and that their responses can be modified through experience. As suggested by the ASL theory, these experiences should be characterized by contingent or predictive relationship between observed and performed actions (Heyes, 2010). Based on this theory it could be hypothesized that repeated and highly predictive experience of being imitated could augment the mirror system response. More studies are needed to support this hypothesis. Currently, behavioral studies had demonstrated that “being imitated” is more efficient than “contingency” in promoting visual attention, imitation recognition and imitation production in children with ASD. At a neural level, the observation of an imitative action in typical children is able to determine a greater desynchronization of the mu rhythm with respect to the observation of a contingent action (Saby et al., 2012). In children with ASD, a reduced mu rhythm desynchronization during movement observation has been repetitively found (Bernier et al., 2007; Oberman and Ramachandran, 2007), but the degree of this reduction is sensitive to the level of familiarity (Oberman et al., 2008). In this light, being imitated might also be a useful tool to assess the neural effects of the interventions for ASD.

Second, the “social motivation theory,” posits that deficits in the social reward system at the neural level alter the way by which children with ASD orient and engage with social stimuli, and consequently impair the social skill development (Dawson, 2008). Indeed, it has been demonstrated that children with ASD have reward anticipation and processing deficits for social stimuli. While typically developing children find social stimuli more salient than nonsocial stimuli, children with ASD may have the opposite preference (Stavropoulos and Carver, 2014). In this framework, the impairment in social attention is considered the cause of the impaired social cognition: based on this theory, some studies reported abnormal activation in the orbito frontal-striatum-amygdala circuit in response to social stimuli (Zeeland et al., 2010; Dichter et al., 2012). It is, in this light, crucial the identification of those strategies that are able to increase the social attention, in particular for early intervention.

Both the brain areas constituting the MNS (such as the inferior parietal cortex) and the neural circuitry related to social reward (such as the ventromedial prefrontal and orbitofrontal cortex) are activated during the “being imitated” condition (Decety and Chaminade, 2003; Kühn et al., 2011). In the first case, the activation was associated with the process of self-other mapping; in the second case, it has been attributed to the social reward arising from sharing the same emotional state or body movement. Thus, the “being imitated” condition could improve some neural circuits that are impaired in ASD and their activation might be useful in improving the clinical outcome of these children, both at the behavioral and biological level.

## Conclusions and future directions

In this review we have discussed findings from behavioral and neuroscientific studies to identify the role of “being imitated” in improving social behavior in children with ASD, one of the most prevalent forms of developmental disability worldwide (Baio, 2012). Some intervention models, indeed, use imitation as a strategy to improve social cognitive skills (Ingersoll, 2008; Rogers et al., 2012).

While the findings of our review show that the imitation of the child's actions could be a tool in early intervention for children with ASD to improve social attention and responsiveness, and play skills, a number of questions remain open. The use of the imitation in improving motor, as well as social abilities in ASD deserves to be tested in future research. Only few studies have investigated whether the positive effect of being imitated on children's social behaviors extends to the imitation skills. They found a slight increase in imitation skills, but more studies are needed given the lack of research on this specific topic.

In addition, the neural basis of being imitated needs to be further investigated both in typically developing children and in those with ASD. Future research could also test the hypothesis that early interventions might result not only in an increase of social skills, but also in a reorganization of neural circuits altered in ASD (Dawson et al., 2002; Dawson, 2008). As the neural bases of imitation have been described, novel electrophysiological or imaging studies could be employed to investigate the neural reorganization of brain networks after intervention, leading to the identification of the most useful strategies for improving brain functioning and behavior.

In conclusion, the “being imitated” in ASD is a key point that could allow to gain new insight into the link between brain, imitation, and social deficits in ASD, and implement more effective intervention strategies, whose effect could be assessed at both behavioral and neural level.

## Author contributions

AC, CC, AN, and FM designed the work, revised, and analyzed critically the literature data, wrote the manuscript, approved the final version, and agreed to its publication.

## Funding

This research was partially supported by grants from the Italian Ministry of Health (Ricerca Corrente to AC and FM).

### Conflict of interest statement

The authors declare that the research was conducted in the absence of any commercial or financial relationships that could be construed as a potential conflict of interest.
